# Misoprostol for treating postpartum haemorrhage: a randomized controlled trial [ISRCTN72263357]

**DOI:** 10.1186/1471-2393-4-16

**Published:** 2004-08-06

**Authors:** G Justus Hofmeyr, Sandra Ferreira, V Cheryl Nikodem, Lindeka Mangesi, Mandisa Singata, Zukiswa Jafta, Babalwa Maholwana, Zonke Mlokoti, Gijs Walraven, A Metin Gülmezoglu

**Affiliations:** 1 Effective Care Research Unit, University of Witwatersrand and Fort Hare, and East London Hospital Complex, East London, South Africa; 2 Department of Nursing, University of the Western Cape, Cape Town, South Africa; 3 Tembisa Hospital Effective Care Research Unit, Tembisa, South Africa; 4 Aga Khan Health Services, Aiglemont, France; 5 HRP/RHR, World Health Organisation, Geneva, Switzerland

## Abstract

**Background:**

Postpartum haemorrhage remains an important cause of maternal death despite treatment with conventional therapy. Uncontrolled studies and one randomised comparison with conventional oxytocics have reported dramatic effects with high-dose misoprostol, usually given rectally, for treatment of postpartum haemorrhage, but this has not been evaluated in a placebo-controlled trial.

**Methods:**

The study was conducted at East London Hospital Complex, Tembisa and Chris Hani Baragwanath Hospitals, South Africa. Routine active management of the third stage of labour was practised. Women with more than usual postpartum bleeding thought to be related to inadequate uterine contraction were invited to participate, and to sign informed consent. All routine treatment was given from a special 'Postpartum Haemorrhage Trolley'. In addition, participants who consented were enrolled by drawing the next in a series of randomised treatment packs containing either misoprostol 5 × 200 μg or similar placebo, which were given 1 orally, 2 sublingually and 2 rectally.

**Results:**

With misoprostol there was a trend to reduced blood loss ≥500 ml in 1 hour after enrolment measured in a flat plastic 'fracture bedpan', the primary outcome (6/117 vs 11/120, relative risk 0.56; 95% confidence interval 0.21 to 1.46). There was no difference in mean blood loss or haemoglobin level on day 1 after birth < 6 g/dl or blood transfusion. Side-effects were increased, namely shivering (63/116 vs 30/118; 2.14, 1.50 to 3.04) and pyrexia > 38.5°C (11/114 vs 2/118; 5.69, 1.29 to 25). In the misoprostol group 3 women underwent hysterectomy of whom 1 died, and there were 2 further maternal deaths.

**Conclusions:**

Because of a lower than expected incidence of the primary outcome in the placebo group, the study was underpowered. We could not confirm the dramatic effect of misoprostol reported in several unblinded studies, but the results do not exclude a clinically important effect. Larger studies are needed to assess substantive outcomes and risks before misoprostol enters routine use.

## Background

Excessive bleeding from the genital tract after birth, or postpartum haemorrhage (PPH) is the major cause of maternal deaths in many low-income countries. The global estimate is 125,000 deaths per year [1]. In South Africa, 240 of 2,445 maternal deaths reported between 1999 and 2001 were due to postpartum haemorrhage, the third most common cause [2]. In a community-based study in Senegal, estimates of maternal mortality ratio in three regions ranged from 436 to 852 per 100,000 live births. Two-thirds were due to direct obstetric causes, the commonest being haemorrhage [3]. In the United Kingdom, the risk of maternal death from haemorrhage is about 1 in 100 000 births [4]. The potential to save mothers' lives with medical interventions for haemorrhage is thus considerable.

There is good evidence that a policy of active management of the third stage of labour [5], and one component of active management, the routine administration of uterotonics (drugs to contract the uerus) such as oxytocin [6] or oxytocin and ergometrine [7], are effective in reducing the risk of postpartum haemorrhage.

When postpartum haemorrhage occurs, a number of medical and surgical interventions are used to control the bleeding [8]. A crucial aspect of both prevention and treatment of postpartum haemorrhage is uterotonic therapy. The most commonly used agents are injectable oxytocin and/or ergometrine.

Misoprostol is a methyl ester (a synthetic analogue) of natural prostaglandin E1. It is marketed and registered for use in the prevention and treatment of peptic ulcer disease caused by prostanglandin synthetase inhibitors. Administered orally or sublingually, peak plasma concentrations of misoprostolic acid are achieved in less than 30 minutes [9]. It is a thermostable drug [9] and is relatively inexpensive.

Administered orally or vaginally, it is an effective agent for the induction of abortion [10] and of labour [11].

### Misoprostol for routine prevention of postpartum haemorrhage

Oral and rectal misoprostol have been used for routine management of the third stage of labour (after the birth). Several small trials have given conflicting results. The main side-effects have been shivering and pyrexia, which are dose-dependent [12]. In the large WHO Collaborative Trial of Misoprostol in the Management of the Third Stage of Labour [13] and a systematic review of the topic [14], blood loss >1,000 ml and use of additional oxytocics (the primary outcomes in the WHO trial) were more frequent with misoprostol than with injectable oxytocics, indicating that injectable oxytocics should remain the drug of choice for routine prophylaxis. On the other hand, blood transfusion was used less frequently in the misoprostol group. This may have been a chance occurrence (it was not a primary outcome for the trial). Secondly, there could be a synergistic pharmacological effect between misoprostol and conventional uterotonics, which were given to most women with increased blood loss. Many women in the misoprostol group thus received misoprostol as well as conventional uterotonics, whereas those in the oxytocin group received only conventional oxytocics. Thirdly, we have suggested in the report of a pharmacokinetic study linked to the WHO trial that the longer time to peak levels of misoprostol (20–30 minutes) than syntocinon (3 minutes) may account for more early blood loss with misoprostol [15]. This does not exclude the possibility that misoprostol may have an effect on more persistent bleeding. Physiological studies have also shown a more rapid onset of uterine contractions following syntometrine than misoprostol after delivery [16].

Misoprostol has been widely recommended for the prevention of postpartum haemorrhage when other methods are not available [17].

### Misoprostol for treatment of postpartum haemorrhage

Apart from the prophylactic use of misoprostol in the third stage of labour, the therapeutic use of misoprostol for the treatment of postpartum haemorrhage has been promoted on the basis of unblinded studies. In a systematic review [18] we located 6 uncontrolled reports (41 women) [19-24] and one small, unblinded randomised trial [25]. This method has entered clinical use, particularly in developing countries, without systematic research to document the optimal route and dosage, effectiveness or risks of this treatment. The urgent need for randomised trials of this new intervention has been emphasised [26]. If effective it could have a major impact on maternal mortality, particulary in under-resourced countries. If not, its use should be discouraged because of the dangers of side-effects, and the risks associated with widespread introduction of misoprostol into health systems in which it might be used for labour induction in inappropriate doses, with the risk of fetal compromise and uterine rupture [27,28].

### Route and dosage of misoprostol

The most common regimens reported for the treatment of postpartum haemorrhage are 800 [23] or 1,000 μg [19,21] rectally. We reviewed the literature on the pharmacokinetics of misoprostol administered by various routes [18]. The oral route has the most rapid uptake, but the shortest duration. The rectal route has slow uptake but prolonged duration. The buccal or sublingual route has rapid uptake, prolonged duration and greatest total bioavailability. In order to improve the rapidity of onset of action and the overall bioavailability, we modified the reported practice of using 1,000 μg rectally, by administering 200 μg orally, 400 μg sublingually and 400 μg rectally.

### Objective

The objective of this study was to determine the side-effects and effectiveness of high-dose misoprostol for the treatment of postpartum haemorrhage by means of a double-blind, placebo-controlled, randomised clinical trial. The hypothesis was that measured blood loss of 500 ml or more in the hour after enrolment would be significantly less frequent in the misoprostol than in the placebo group.

## Methods

The study was conducted at the East London Hospital Complex (Frere Maternity and Cecilia Makiwane Hospitals, Eastern Cape) and Tembisa and Chris Hani Baragwanath Hospitals, Gauteng, South Africa, between Jan 2002 and Dec 2003. All are busy referral hospitals.

Treatment packs were prepared independently, ordered in computer-generated random sequence and numbered consecutively. The packs contained either misoprostol 5 × 200 μg or inactive placebo.

The treatment sequence was kept sealed, and the code broken only after complete entry and checking of all trial data.

A confidential interim analysis, blind to which group was which, was performed by AMG after the first 100 enrolments. Evidence of clear benefit or harm for either group would have prompted stopping the trial, which was not the case.

Women in labour were given basic information about the trial in the form of notices in the labour rooms (Table 2 [see additional file 1 ]). Management of labour 3^rd ^stage was routine active management with oxytocin 10 units or syntometrine one ampoule soon after the birth. Women aged 18 or more with bleeding judged by the clinician to be more than expected at least 10 minutes after delivery, and thought to be due to uterine atony and requiring additional uterotonic therapy, were given all the routine treatment for PPH (intravenous infusion, uterotonics, etc) from a special 'PPH Trolley' kept in the labour ward. The uterotonics used were oxytocin by intravenous infusion, and/or oxytocin/ergometrine, at the discretion of the attending clinician. Prostaglandin preparations such as sulprostone were not routinely available in the labour wards, and were not part of routine management of postpartum haemorrhage in the participating units.

Once all emergency treatment was instituted, and if the women were in a position to give fully informed consent, they were given detailed information about the trial and asked whether they wished to participate. Information about the trial was given verbally and in writing in English or Xhosa (Table 3 [see additional file 2 ]). Those who gave written consent were enrolled in the trial. Baseline data were documented. The next treatment pack containing either misoprostol 5 × 200 μg or inactive placebo was drawn and the number recorded. In addition to routine management, misoprostol or placebo (an inactive base) were given, 1 orally, 2 buccally/sublingually and 2 rectally, and the time recorded.

A low-profile plastic 'fracture bedpan' was placed under the woman's buttocks. In previous studies we have shown this to be an efficient method of collecting ongoing blood loss with very little spillage [29]. Any small swabs soaked with blood were dropped into the bedpan. After 1 hour, the blood collected in the bedpan was measured in a graduated measuring jug, the temperature was measured sublingually with a mercury thermometer, and any shivering was subjectively recorded as 'mild' (not causing any distress), 'moderate' or 'severe'.

All other management was by hospital staff according to the hospital routine for the management of postpartum haemorrhage.

After 24 hours, blood was collected for haemoglobin estimation and the hospital records checked for use of additional uterotonics and any other complications such as blood transfusion.

The primary outcome measures were specified prior to commencing the study: (1) measured blood loss ≥500 ml in 1 hour after enrolment; (2) mean measured blood loss in 1 hour after enrolment; (3) haemoglobin level day 1 after birth <6 g/dl or blood transfusion; (4) Side-effects (pyrexia 38.5°C or more, moderate or severe shivering 1 hour after enrolment).

Secondary outcome measures were: (1) blood loss ≥1,000 ml in 1 hour after enrolment; (2) blood transfusion; (3) haemoglobin level 1 day after birth <8 g/dl or blood transfusion; (4) additional uterotonic given after enrolment; (5) manual removal of the placenta; (6) evacuation of retained products of conception; (7) hysterectomy; (8) maternal death.

### Sample size calculation

In the WHO misoprostol trial control group (routine syntocinon), significant postpartum haemorrhage (blood loss >1000 ml) occurred in 2.8% of women. Of these 25% lost >1,500 ml. To reduce blood loss >500 ml after enrolment from 25% to 10%, would require 112 per group (α = 95%, β = 80%).

Data were collected on a data form, entered onto an excel spreadsheet, checked for accuracy, then analysed using Epi-info 2002 (United States Department of Health and Human Services, Centres for Diseases Control and Prevention, Epidemiology Program Office) and Review Manager (RevMan) [Computer program] version 4.2 for Windows. Oxford, England: The Cochrane Collaboration, 2003. Results were expressed as relative risks or mean difference with 95% confidence intervals. Analysis was by intention to treat.

### Ethical considerations

All women enrolled in the trial received all the conventional management available for postpartum haemorrhage, in addition to the trial medication (misoprostol or placebo). Rapid conventional therapy was facilitated by the ready availability of the 'PPH trolley' with all the necessary materials. This trolley was also available for women not enrolled in the trial. Participation was limited to women who were in a position to give fully informed consent. Ethical approval was given by the Committee for Research on Human Subjects, University of the Witwatersrand, and the Ethics Committee, East London Hospital Complex. The trial complied with the Helsinki Declaration for research on human subjects.

## Results

Altogether 244 women were enrolled in the trial. The pack numbers for 6 women were incompletely filled in on the data sheets. The group allocation of these women was therefore unknown, and they could not be included in the analysis. Of these 6 women, the highest measured blood loss after enrolment was 220 ml; none had pyrexia >37.5°C, one had moderate shivering, one had a blood transfusion and none had day 1 haemoglobin level below 8 g/dl or other complications. There was no reason to expect that these exclusions occurred in any selective way which would introduce bias into the final samples, nor could their results have materially altered the conclusions of the study. All the remaining 238 women were included in the analyses.

The baseline characteristics are shown in table 1. As would be expected in a randomised trial, the groups were well matched. Parity was not recorded at all hospitals, but was similar between groups where recorded (misoprostol mean 1.61, SD 1.14; placebo 1.75, SD 1.26).

**Table 1:** Comparison of outcomes between women who received misoprostol or placebo, expressed as proportions (percent) or mean values [standard deviation]. Differences are expressed as relative risks or mean differences, with 95% confidence intervals.

	Misoprostol (117)		Placebo (121)			
		
Baseline characteristics	n		n			
Age (years)	116	27.1 [6.0]	119	26.2 [6.2]		
Ergometrine before enrolment	106	36 (34%)	104	34 (33%)		
Oxytocin ≥20 U before enrolment	116	82 (71%)	117	78 (67%)		
Primary outcomes:					RR/ MD	95% Conf. interval
Blood loss ≥500 ml*	117	6 (5.1%)	120	11 (9.2%)	0.56	0.21 to 1.46
Mean blood loss* [SD] (ml)	117	168 [163]	120	176 [173]	-8	-51 to 35
24 hour Haemoglobin <6 g/dL or blood transfusion	110	20 (18.2%)	116	17 (14.7%)	1.24	0.69 to 2.24
Pyrexia at 1 hour ≥38, 5°C	114	11 (9.6%)	118	2 (1.7%)	5.69	1.29 to 25
Shivering at 1 hour ≥ moderate	116	63 (54%)	118	30 (25%)	2.14	1.50 to 3.04
Secondary outcomes:						
Blood loss ≥ 1,000 ml*	117	1 (0.85%)	120	0 (0%)		
Blood transfusion	115	19 (17%)	119	15 (13%)	1.31	0.70 to 2.45
24 hour haemoglobin <8 g/dL or blood transfusion	110	43 (39%)	116	37 (32%)	1.23	0.86 to 1.75
Additional uterotonic after enrolment	111	63 (57%)	112	63 (56%)	1.01	0.80 to 1.27
Manual removal of the placenta	117	1 (0.85%)	121	4 (3.3%)	0.26	0.03 to 2.28
Evacuation of retained products of conception	117	2 (1.7%)	121	0 (0%)		
Hysterectomy**	117	3 (2.6%)	121	0 (0%)		
Maternal death**	117	3 (2.6%)	121	0 (0%)		

RR = relative risk; MD = mean difference; Conf = confidence; SD = standard deviation *Measured, within 1 hour after enrolment ** One woman died after hysterectomy and is counted in both outcomes

The outcomes are shown in table 1. In the placebo group, the primary outcome (measured blood loss within 1 hour after enrolment of 500 ml or more) was less frequent (9.2%) than the 25% prediction on which the sample size calculation was based. This may have been because the criteria for enrolment were loosely defined as 'more than expected blood loss', resulting in the enrolment of women with less severe blood loss than anticipated. The fact that enrolment was limited to women who were able to received detailed information about the study, may have caused selection of less severe cases. Although the intention was to enrol women who had not responded to conventional therapy, the time taken for counselling of the women and obtaining informed consent may have resulted in some women responding to the primary treatment, and therefore the low rate of ongoing blood loss in the placebo group. The study was thus underpowered to detect a statistically significant reduction in the primary outcome. With misoprostol there was a trend to reduced blood loss ≥500 ml in 1 hour after enrolment (6/117 vs 11/120, relative risk 0.56; 95% confidence interval 0.21 to 1.46).

Other proxy estimations of blood loss showed no significant differences between the groups. The well-known side-effects of misoprostol, pyrexia and shivering, were significantly more frequent in the misoprostol than the placebo group.

Serious morbidity or mortality occurred in 5 women, all from the misoprostol group. Two women who continued to bleed despite conservative therapy underwent abdominal hysterectomy and recovered well. There were 3 maternal deaths:

### Case 1

A 22-year old woman with one previous pregnancy developed postpartum haemorrhage after vaginal delivery, managed with a 40-unit oxytocin infusion. She was enrolled and received the trial medication 85 minutes after delivery. Thereafter she received a second 40-unit oxytocin drip, oxytocin/ergometrine (5 units/0.5 mg) and intravenous cyclokapron. In the hour after randomisation, measured blood loss was 125 ml. Subsequently bleeding continued and a sub-total hysterectomy was performed. Coagulopathy developed, bleeding continued through an abdominal drain, and she died 2 days after delivery despite re-laparotomy and multiple blood product transfusions.

### Case 2

A 32-year-old woman, para 2, gravida 4, delivered normally after labour was induced with misoprostol, 25 μg vaginally and 4 doses of 50 μg orally. Before enrolment she received oxytocin10 units, ergometrine 0.5 mg and a 20-unit oxytocin infusion. She was enrolled and received the trial medication 140 minutes after delivery. After enrolment she received a further 40-unit oxytocin infusion. Measured blood loss in the hour after enrolment was 380 ml. Bleeding continued and a blood transfusion was commenced. Six and a half hours after delivery she suffered a cardiac arrest and was resuscitated. Examination in theatre found the uterus to be empty and intact. The main source of bleeding appeared to be a torn cervix, which was sutured. At 7.5 hours after delivery she suffered a second cardiac arrest and could not be resuscitated. The estimated total blood loss was 3,000 ml.

### Case 3

A woman with one previous delivery by caesarean section, developed postpartum haemorrhage of about 500 ml after a vaginal delivery. She was enrolled 85 minutes after delivery. Measured blood loss over the next hour was 425 ml, after which she developed hypotension and cardiorespiratory arrest. As no post-mortem examination was performed, the possibility of internal bleeding from a dehisced caesarean section scar could neither be confirmed nor excluded.

Unresponsive uterine atony was therefore not confirmed as the main cause of any of the deaths.

## Discussion

Pyrexia and shivering were common side-effects of misoprostol, as found in previous studies. Three women, all in the misoprostol group, had severe pyrexia >40°C. Previous studies of high-dose misoprostol for the treatment of postpartum haemorrhage have mostly used the rectal route, and none has had sufficient numbers to be likely to detect rare adverse outcomes such as severe pyrexia. These side-effects may be related to the rapid absorption of misoprostol given orally, and the rapid absorption and high bioavailability when given sublingually. Because of these serious side-effects, we would recommend that for future trials the dose be reduced.

Several previous unblinded studies have reported dramatic effects of misoprostol when used to treat postpartum haemorrhage. In 6 observational studies [19-24], 41 women with postpartum haemorrhage were treated with misoprostol 1,000 μg rectally (32 women), 200 μg rectally (5 women), 200 μg orally 2-hourly (2 women) or 800 μg intrauterine (2 women). In all but 2 of the women (who received 1,000 μg rectally), a prompt response to the treatment was reported. In a single blind randomised trial [25] subjective prompt cessation of bleeding was reported in 30/32 women who received misoprostol 800 μg rectally compared with only 21/32 who received oxytocin 5 units plus ergometrine 0.5 mg intramuscularly.

## Conclusions

In our double blind trial we have been unable to confirm the dramatic effects of misoprostol reported previously in unblinded studies. Our results, however, are consistent with anything from a large beneficial effect to a smaller adverse effect (relative risk of additional blood loss of 500 ml or more anywhere between a reduction of 79% and an increase of 46% with misoprostol). It is important that this possible benefivial effect be assessed by sufficiently powered randomised trials before the treatment enters routine clinical use.

## List of abbreviations

PPH: postpartum haemorrhage; SD: standard deviation

## Competing interests

None known.

## Authors' contributions

GJH prepared the first draft of the protocol and the final manuscript, and oversaw the clinical work

CN assisted with the design of the protocol and data collection sheet, initiated enrolment at two sites, supervised some of the data collection and contributed to the manuscript

SF, ZM, LM, MS and ZJ commented on the protocol, conducted the clinical procedures and collected data.

SF entered and collated the data

BM undertook the literature search and commented on the protocol.

GW contributed to the protocol development

AMG contributed to the protocol development and undertook the blinded interim analysis

All authors read and approved the final manuscript

## Additional files

Additional file 1 - Table 2. Notice informing women about the trial, MS Word file: HofmeyrFile1.doc

Additional file 2- Table 3: Information and consent form. MS Word file: HofmeyrFile2.doc

## Pre-publication history

The pre-publication history for this paper can be accessed here:



## Supplementary Material

Additional File 1 Table 2. Notice informing women about the trial Click here for file

Additional File 2 Table 3: Information and consent form Click here for file
