# Supplementary material for: Misoprostol for treating postpartum haemorrhage: a randomized controlled trial [ISRCTN72263357]
Source: BMC Pregnancy Childbirth. 2004 Aug 6;4:16. doi: 10.1186/1471-2393-4-16 (PMC514549; doi:10.1186/1471-2393-4-16)
Supplement: Additional File 1 — Table 2. Notice informing women about the trial [file 1471-2393-4-16-S1.doc]

**Table 1. Notice informing women about the trial (Additional file 1)**

After the birth,

there is usually a little bleeding

If the bleeding is too much,

we will ask you to join a research study

You will be free to say ‘yes’ or ‘no’

If you say ‘yes’, we will give you

extra tablets to stop the bleeding

The tablets may help, or may not help

The results of the study

will help other women
