# Supplementary material for: Misoprostol for treating postpartum haemorrhage: a randomized controlled trial [ISRCTN72263357]
Source: BMC Pregnancy Childbirth. 2004 Aug 6;4:16. doi: 10.1186/1471-2393-4-16 (PMC514549; doi:10.1186/1471-2393-4-16)
Supplement: Additional File 2 — Table 3: Information and consent form [file 1471-2393-4-16-S2.doc]

# Table 2: Information and consent form (Additional file 2)

# Province of the Eastern Cape, Isebe lezempilo/Department of Health

**East London Hospital Complex**

**Effective Care Research Unit, University of the Witwatersrand.**

**Information for women and Informed consent form**

**Misoprostol for treating postpartum haemorrhage**

The purpose of the study: After birth, most women have mild bleeding, but a few women have heavy bleeding. The treatments available do not always work completely. This study is to find out whether a new tablet in addition to the usual treatment will be more effective in stopping the bleeding.

# Procedures: If you agree to participate, your bleeding will as usual be checked after the birth. If it seems to be more than usual, we will give all the normal treatment, and in addition give one tablet orally, two tablets sublingually (under the tongue) and two tablets rectally (back passage). For some women in the trial these tablets will be misoprostol, the new tablet we are testing. For others, it will be an inactive tablet. We will carefully measure all the blood loss in a bedpan, and give all the treatment needed.

Risks & benefits: The benefits are that all women in the trial will quickly get all the necessary treatment. Those who also get the misoprostol may have further benefit, but this has not yet been proved. The risk is that the misoprostol may cause some side-effects. The known side effects are occasional shivering or fever, and ‘stomach’ upset.

Rights;

Your involvement in this study is entirely voluntary, and if you decide not to take part, it will not affect the medical treatment you get in any way. If you decide to join the study, you are free to leave the study at any time without explanation.

Confidentiality:

All the records will be kept in a safe place and your name will not be used

Contacts:

The research midwife or doctor will leave a contact phone number for you, which you can call if you need more information.

Consent

**This explanation of the study was given by ……………………………….. on**

the ^D D M M Y Y Y Y^. Signature of person explaining…………………

Name of participant ………………………………………..

I agree to take part in this study and understand that my participation is voluntary, may be withdrawn at any time, and will not affect my medical treatment in any way.

Signature ……………………. …………… Date ^D D M M Y Y Y Y^

Signature of Witness if thumbprint or if interpreter used.

Witness name………………………Witness signature……………………………….
